# A flexible and economical barcoding approach for highly multiplexed amplicon sequencing of diverse target genes

**DOI:** 10.3389/fmicb.2015.00731

**Published:** 2015-07-16

**Authors:** Craig W. Herbold, Claus Pelikan, Orest Kuzyk, Bela Hausmann, Roey Angel, David Berry, Alexander Loy

**Affiliations:** Division of Microbial Ecology, Department of Microbiology and Ecosystem Science, Research Network Chemistry Meets Microbiology, University of ViennaVienna, Austria

**Keywords:** MiSeq, functional gene, 16S rRNA, amoA, nifH, dsrA, dsrB, nxrB

## Abstract

High throughput sequencing of phylogenetic and functional gene amplicons provides tremendous insight into the structure and functional potential of complex microbial communities. Here, we introduce a highly adaptable and economical PCR approach to barcoding and pooling libraries of numerous target genes. In this approach, we replace gene- and sequencing platform-specific fusion primers with general, interchangeable barcoding primers, enabling nearly limitless customized barcode-primer combinations. Compared to barcoding with long fusion primers, our multiple-target gene approach is more economical because it overall requires lower number of primers and is based on short primers with generally lower synthesis and purification costs. To highlight our approach, we pooled over 900 different small-subunit rRNA and functional gene amplicon libraries obtained from various environmental or host-associated microbial community samples into a single, paired-end Illumina MiSeq run. Although the amplicon regions ranged in size from approximately 290 to 720 bp, we found no significant systematic sequencing bias related to amplicon length or gene target. Our results indicate that this flexible multiplexing approach produces large, diverse, and high quality sets of amplicon sequence data for modern studies in microbial ecology.

## Introduction

The gold standard for analyzing and comparing microbial communities across many environmental or medical samples is met with high throughput sequencing of 16S rRNA and functional marker gene amplicons. Current sequencing technologies enable the generation of millions of reads per run, and parallel sequencing of multiple samples can be accomplished through the introduction of a sample-specific short sequence tag (barcode, index) at one or both ends of the target gene amplicon during library preparation. This so-called barcoding or indexing is commonly achieved by performing PCR with large (>50 nucleotide) fusion primers that consist of the original gene-specific primer, linkers, barcodes, and sequencing platform-specific adapter sequences (Sogin et al., 2006; Fadrosh et al., 2014). Single-step PCR with long fusion primers can lead to differences in amplification efficiency and accuracy between samples, a problem which can be ameliorated to some extent with a two-step PCR procedure in which the large fusion primers are added to PCR amplicon products via a second PCR with a low number of cycles (Berry et al., 2011). Regardless of general strategy (one-step vs. two-step), PCR-based barcoding procedures using large fusion primers can become prohibitively expensive when a study includes many samples and different gene targets are analyzed, each of which requires a specific barcoded fusion primer. Hence, new barcoding approaches need to be established to alleviate this burden (Kozich et al., 2013; Fadrosh et al., 2014).

**Figure 1:**
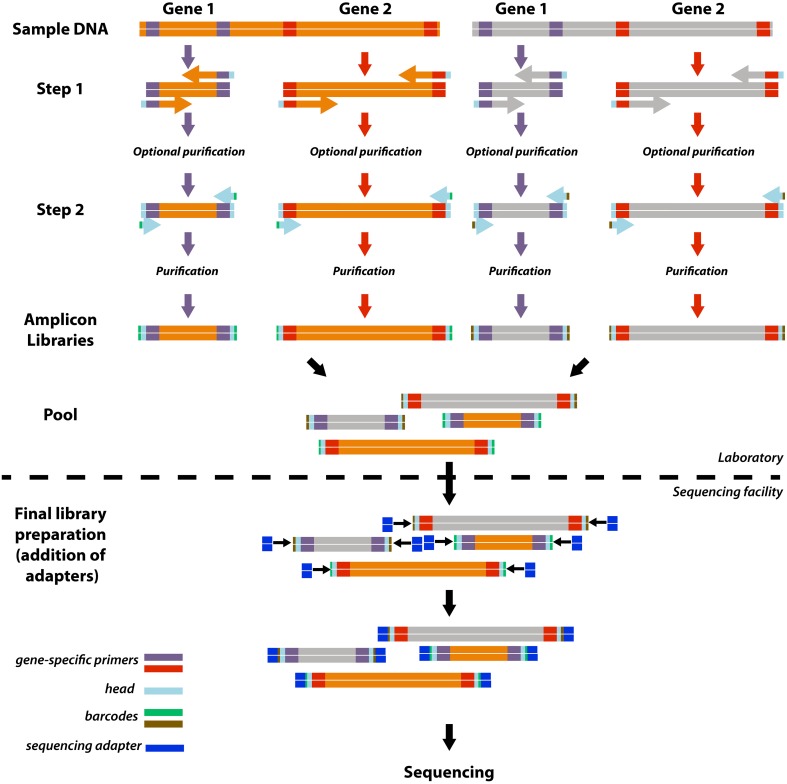
**Schematic overview of the approach**. For simplification this schematic shows how four amplicon libraries are produced from two gene targets and two samples. The approach includes a 2-step PCR with gene target-head primers in the first step and head-barcode primers in the second step. Reactions for each gene target/sample combination are carried out independently and all libraries with a unique combination of barcode and primer may be combined to produce a single pooled library for sequencing. Further library preparations, including attachment of the sequencing platform-specific adaptor sequences, may be outsourced to sequencing facilities.

Here, we developed a simple, highly adaptable, and cost-effective version of the two-step PCR barcoding approach that enables the efficient construction of a customized sequencing library of multiple gene targets from various samples (Figure 1). In our approach, the first PCR step introduces a universal 16 bp head sequence (5′- *HEAD*-TARGET PRIMER-3′). The amplicon is then tagged during a second step of PCR with a primer that targets only the head sequence but also encodes an 8 bp barcode (5′-BARCODE-*HEAD*-3′). With this two-step PCR approach a universal set of barcode-head primers can be used repeatedly for barcoding diverse amplicons of interest, and thus costly investment into individual barcoded primer sets for each target gene is not required. Subsequently, the pooled library of multiple amplicons can be adapted to any sequencing platform by introducing the appropriate sequencing adapters during library preparation (Figure 1).

## Materials and methods

### Mock communities

Two mock communities were used to evaluate amplicon preparation, data quality, and quantitative biases. Both communities consisted of the same five 16S rRNA gene clones (H42, AF234715; H29, AF234692; H28, AF234749; H13, AF234737; H44, AF234743) from an activated sludge study (Juretschko et al., 2002) that were combined to have even or uneven proportions. Purified plasmids were quantified using the Qubit® dsDNA BR Assay Kit (Life Technologies) and a Qubit® 2.0 Fluorometer system (Life Technologies). For the even mock community the plasmids were mixed in equimolar proportions, for the uneven mock community the clones were mixed in a more realistic fashion, resulting in relative abundances of the individual clones at 76, 18, 5, 0.7, and 0.09%, respectively. After construction, the even and uneven mock communities were diluted to a final concentration of 0.1 ng μL^−1^.

### PCR

Amplification was performed with a two-step barcoding approach (Figure 1). In a first PCR, target genes were amplified with diagnostic primers (Supplementary Information 1, Table S1.1) synthesized with a 16 bp head sequence [5′-GCTATGCGCGAGCTGC-3′, modified from Rudi et al. (2003)] at the 5′ end. In a second PCR, products were amplified with primers that consist of the 16 bp head sequence and include at the 5′ end a library-specific 8 bp barcode from a previously published list [Hamady et al., 2008, Supplementary Information 1, Table S1.2]. Each PCR reaction (20 μL in first step, 50 μL in second step) consisted of 1 × Taq buffer (Fermentas), 0.2 mM dNTPmix (Fermentas), 2 mM MgCl_2_ (Fermentas), 0.025 U Taq DNA polymerase (Fermentas), 0.1 mg mL^−1^ bovine serum albumin, 1 μM of each of the forward and reverse primers and 1 μL of template. Primers and corresponding annealing temperature for step 1 are all given in Supplementary Information 1, Table S1.1. Thermal cycle conditions were 95°C for 3 min; 95°C for 30 s, primer-specific annealing temperature (step1) or 52°C (step 2) for 30 s, 72°C for 1 min; and 72°C for 7 min. The first PCR reaction was performed in triplicate, screened by gel electrophoresis, and pooled for use as a template in the second step, which used only one primer (5′-BARCODE-*HEAD*-3′). Second step PCR products were also screened by gel electrophoresis. In order to test for specific biases introduced by the two step barcoding procedure, mock communities underwent nine different cycle combinations of first: second cycle number (10:20, 15:15, 20:10, 25:5, 25:10, 30:10, 30:5, 35:10, and 35:10). The barcodes that were used for amplification of the mock community libraries under these conditions are listed in Supplementary Information 1, Table S1.3.

### Purification, quantification, and sequencing

The barcoded amplicons were purified with Agencourt AMPure beads (Beckman Coulter Genomics) and quantified using the Quant-iT™ PicoGreen® dsDNA Assay (Invitrogen). For obtaining a similar number of sequences for each amplicon library, an equimolar library was constructed containing 20 × 10^9^ molecules per individual amplicon library, however amplicons with approximate length > 600 were spiked in twice. The final pooled library (20 ng/μL in 100 μLTris buffer pH 8) was then sent to Microsynth AG (Balgach, Switzerland) for sequencing on a MiSeq system (Illumina). The library was prepared by adaptor ligation and PCR using the TruSeq Nano DNA Library Prep Kit (Illumina, Cat FC-121-4001) according to the TruSeq nano protocol (Illumina, FC-121-4003), but excluding the fragmentation step. The library was quantified by qPCR (Kapa Biosystems). Illumina sequencing requires high base variability during the first cycles (the first five bases are most critical) for efficient sequence cluster identification and phasing/pre-phasing calibration (Fadrosh et al., 2014). Here, we achieve higher sequence variability during the first cycles by using approximately 300 barcodes with heterogeneous base composition and by spiking in a random shotgun library (instead of PhiX) at 10% abundance. The MiSeq was run in the 2 × 300 cycle configuration using the MiSeq Reagent kit v3 (Illumina, Cat MS-102-3003). Sequencing adaptors were removed from reads and the random shotgun library was filtered from the dataset by Microsynth. Resulting datasets were deposited in the NCBI Sequence Read Archive under study accession number SRP059317.

### Sequence processing and analysis

**Figure 2:**
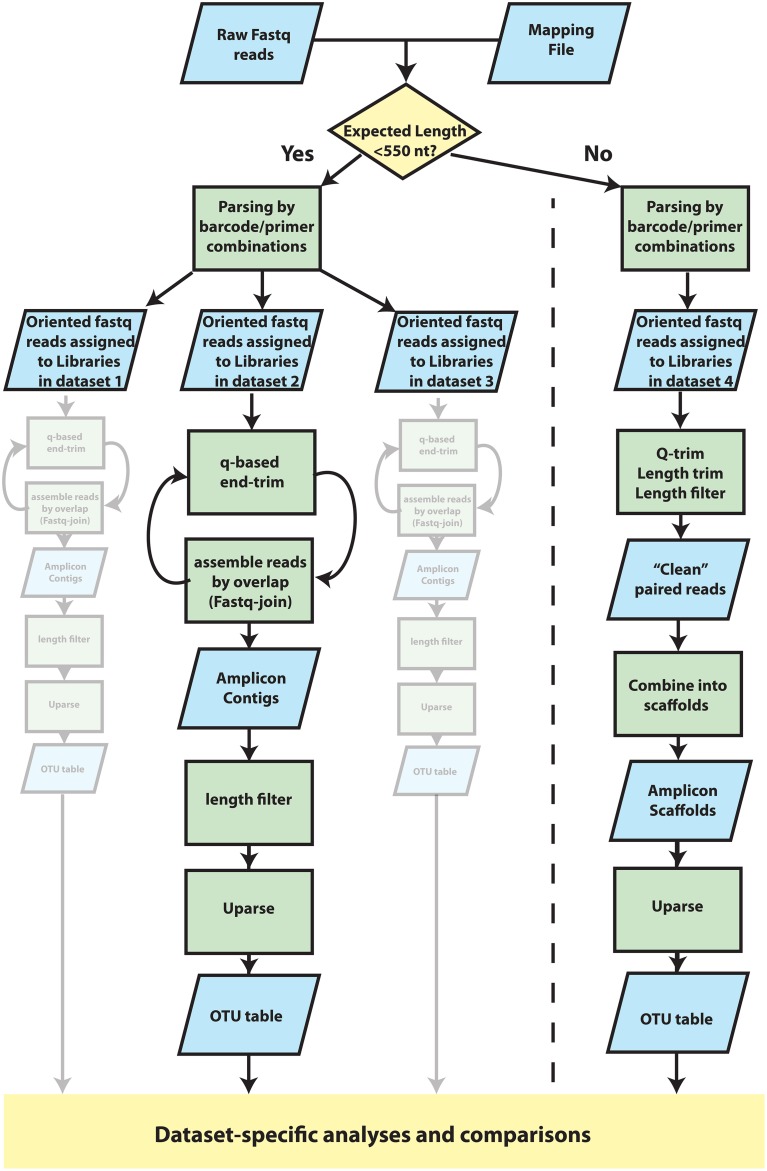
**Overview of procedures that were used to cluster reads into Operational Taxonomic Units (OTUs)**. Detailed methods are provided in the main text. Fastq data was parsed into datasets by two analysis paths according to whether the expected amplicon length was less than or greater than 550 nucleotides in length. In both paths, reads were oriented and assigned to libraries within datasets according to a specific combination of barcode/primer. Each dataset was then filtered and processed in parallel and independently of one another. For datasets in which the expected amplicon length was less than 550 nucleotides, amplicons were assembled into contigs using an iteration between Q-score-based end-trimming and overlap-based assembly. Short contigs (<75% of expected amplicon length) were then discarded before clustering into OTUs with UPARSE. For datasets of long (>550 nt) amplicon targets, for which little if any overlap was expected, reads were instead combined into oriented, alignable scaffolds. Reads were end trimmed according to Q-score thresholds and length. Read pairs were removed if either read was less than 75% of the original read length after trimming, which resulted in individual reads with uniform length. Forward reads were then concatenated to the reverse complement of the reverse read using a small, arbitrary spacer of 4 ambiguous bases “N” and were subsequently clustered into OTUs using UPARSE. OTU tables were then used for dataset-specific analyses.

Paired reads generated by the MiSeq platform were assembled into OTUs according to the schema presented in Figure 2. We refer to “Library” as a distinct set of sequences that belong to a single observation. A “Dataset” is a group of amplicon libraries that will be analyzed together. First, read pairs were assigned to datasets using an in-house Python script. To be assigned to a library within a dataset, reads were required to match the correct barcode and primer in at least one read and the corresponding primer in the second read. One mismatch was allowed in each barcode and primer sequence examined. Read pairs were then exported into dataset-specific, oriented files. Primer and barcode fastq files were also generated at this step, using corrected barcodes and primers, to facilitate incorporation of the datasets into a QIIME (Caporaso et al., 2010) pipeline. Fastq reads within each dataset were assigned to amplicon libraries using QIIME's split_libraries_fastq.py. Paired-end reads were then collapsed into a single continuous sequence by two different strategies, depending on target amplicon length. For targets under 550 bp, Q-scores were used to end-trim reads, which were then assembled into contigs with join_paired_ends.py with default joining method (fastq-join). A range of Q-scores (0, 3, 10, 15, 20, 25, 30, 35, 36, 37, 38) were used for end-trimming each dataset and the Q-score for end-trimming was chosen based on maximizing the number of contigs produced for that dataset. Any contigs with a length <75% of the dataset-specific approximate amplicon length were discarded. Gene targets over 550 bp underwent quality filtering and trimming using a strategy based on the protocol for Illumina data in the Earth Microbiome Project (Version 5 2012, Gilbert et al., 2014) Reads were end-trimmed to the first instance of a Q-score of 3 or less. If one or both reads in a read pair was shorter than 75% of the original read length after end-trimming, both reads were discarded. All remaining reads were trimmed to 75% of the original length which resulted in datasets of trimmed reads of equal length. After trimming and filtering, read pairs were assembled into a scaffold using an artificial separation of 4 N characters between the forward read and the reverse-complement of the reverse read. Datasets were chimera-checked and clustered using a UPARSE pipeline (Edgar, 2010, 2013). First contigs/scaffolds were dereplicated with the -derep_fullength command and singleton unique sequences were removed. OTU centroids were then determined with the -cluster_otus command (-leftjust -rightjust -maxrejects 0 -maxaccepts 0) and otu_radius was set specific for amplicon (3 for mock communities discussed in text). Abundances of OTUs were determined by mapping the filtered contigs/scaffolds (prior to dereplication) to OTU centroids using the -usearch_global command (-leftjust -rightjust -maxrejects 0 -maxaccepts 0 -maxhits 1) and the -id parameter was set specific to dataset (0.97 for mock communities discussed in text). OTUs were identified as mock community members using a 97% identity threshold in USEARCH. Contaminants were defined as OTU centroids that shared higher identity to non-mock community sequences in co-sequenced datasets or identified through a NCBI blastn search against the nr/nt database (Supplemental Table S2.1). Modeling and statistical comparisons were carried out using lm(), aov(), and TukeyHSD() in the R statistical package as well as vegdist() and mantel() functions from the vegan library (Oksanen et al., 2012).

## Results and discussion

**Figure 3:**
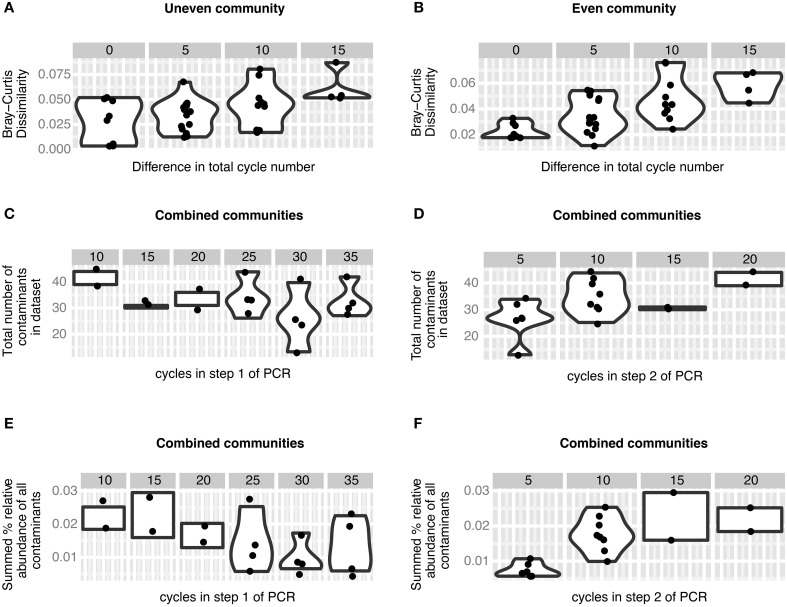
**Effect of PCR cycle number on mock communities. (A,B)** Bray-Curtis dissimilarity increases significantly with increasing differences in number of total PCR cycles (cycle 1 + cycle 2) in the contaminant-free uneven (**A**, *p* = 0.0041) and even (**B**, *p* = 8.7e-06) mock communities. **(C,D)** The number of contaminants observed in the mock community datasets as a response variable in multiple regression does not change significantly with the number of cycles in step 1 of the two-step PCR procedure (**C**, *p* = 0.3) but increases with the number of cycles in step 2 of the two-step PCR procedure (**D**, *p* = 0.008). **(E,F)** The relative abundance of contaminants observed in the mock community datasets as a response variable in multiple regression does not change significantly with the number of cycles in step 1 of the two-step PCR procedure (**E**, *p* = 0.56), but increases with the number of cycles in step 2 of the two-step PCR procedure (**F**, *p* = 0.03).

**Figure 4:**
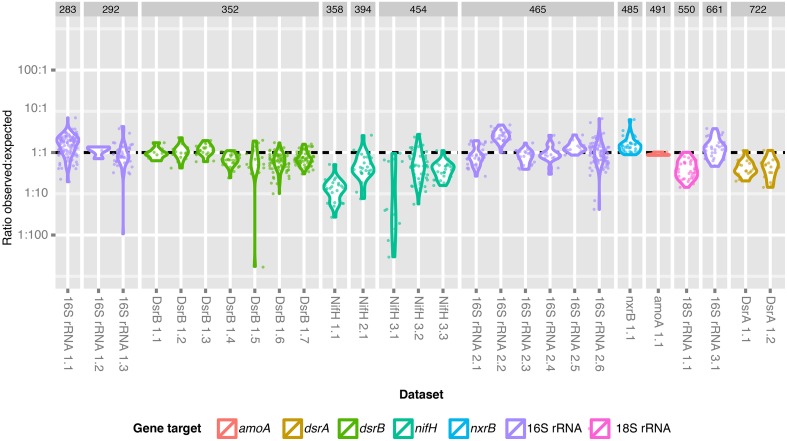
**Ratio of the observed and expected number of sequences for each dataset**. The observed: expected fraction of the library is shown for each dataset and is grouped according to expected amplicon length and gene fragment target. A dashed line indicates the ratio at which expected library fraction = observed library fraction.

To evaluate whether our barcoding and multiplexing strategy can be confidently applied across gene target and biological sample type, we pooled 919 amplicon libraries from 27 datasets (Supplemental Table 2.2) into a single paired-end sequencing run using the Illumina MiSeq platform. We used amplicon libraries of simple mock communities to assess the effect of cycle variation on contamination and inferred community structure (Figure 3) and amplicon libraries from environmental datasets to test whether amplicon yield suffers specific bias under normal operating conditions (Figure 4). Amplicon libraries were constructed with PCR primers targeting archaeal/bacterial and eukaryotic small subunit ribosomal RNA (SSU-rRNA) and functional genes (*amoA*, *dsrA*, *dsrB*, *nifH*, and *nxrB*) of various microbial guilds, using DNA extracted from different biological and ecological samples (mouse intestinal lumen, peatland soil, lake, and marine sediments, cooling tower, and drinking waters) by numerous personnel and pooled. Mock community libraries were constructed from a restricted set of bacterial SSU-rRNA clones from an activated sludge study (Juretschko et al., 2002). These clones were chosen because the sequencing run did not contain any sequence data from similar environments. Final library preparation, paired-end MiSeq sequencing (2 × 300 cycle configuration) and automatic filtering of extremely low quality reads was performed by Microsynth AG (Balgach, Switzerland). The returned cohort of data consisted of 9,989,751 read pairs, of which 7,371,023 (73.7%) were unambiguously assigned to datasets [median per dataset = 6229, 95% CI (771, 24323)]. After quality filtering and assembly/scaffolding of paired reads, we retained 4,288,723 contigs/scaffolds [median per dataset = 3751, 95% CI (48,813,574)]. The proportion of data that passed through our assignment and quality control procedure (42.9%) is consistent with previous reports for the MiSeq platform (e.g., 33.4% Caporaso et al., 2012).

16S rRNA gene amplicons of two simple mock communities, each consisting of five clones, were included to evaluate the influence of our barcoding approach on recoverable community structure. One mock community was constructed unevenly, with individual sequences ranging from 0.09 to 76% relative abundance, and a second community was “even,” with all sequences combined in equimolar proportions. We tested different combinations of cycle number in the first and second PCR step on each mock community and found that mock datasets produced with similar total cycle numbers are more similar to one another than communities with differing numbers of cycles (Figure 3). For both the uneven and even community structure, the Bray-Curtis dissimilarity between different libraries was affected by the total (step 1 + step 2) number of cycles (Mantel test. uneven: *r* = 0.4669, *p* = 0.004. even: *r* = 0.6675, *p* = 8.7e–06), which suggests that the native community structure is distorted in proportion to the number of total PCR cycles. A similar result came from a comparison of correlated abundances across cycle conditions (Mantel test. uneven: *r* = 0.5919, *p* = 0.004. even: *r* = 0.5387, *p* = 0.016). We therefore recommend to keep the number of total cycles as low as reasonably achievable, as others have done to reduce chimera formation, error rates, and kinetic bias (Suzuki et al., 1998; Acinas et al., 2005).

As with any barcoding approach (Esling et al., 2015), our two-step library preparation procedure is sensitive to contamination. For the mock communities we defined an OTU as contamination if its sequence had a higher similarity either to an OTU from the non-mock datasets within our experiment or to a sequence found within Genbank (Supplementary Information 2, Table S2.1). Using this criteria we were surprised to find that nearly all non-mock community OTUs were more likely the result of contamination and not from chimera formation or misinterpretation of sequencing error as novel OTUs (Supplementary Information 1, Figure S1.1). The number of contaminant OTUs observed in our mock communities increased with the number of PCR cycles in step 2 (Figure 3, 1.61 contaminants/cycle, *p* = 0.008) and total read depth (0.0014 contaminants/read, *p* = 0.030), whereas the number of PCR cycles in step 1 (*p* = 0.3) and expected community structure (*p* = 0.33) did not have a significant effect (model adjusted R-squared = 0.412, *p* = 0.025). Likewise, the total proportion of contamination also increased relative to the number of cycles in step 2 (Figure 3, 0.11% contaminant/cycle, *p* = 0.030), whereas the number of cycles in step 1 (*p* = 0.56), total read depth (*p* = 0.14) and expected community structure (*p* = 0.75) had no effect (model adjusted R-squared = 0.497, *p* = 0.01). The abundance of individual contaminants tended to be quite low (median = 0.027%), and covered a broad range of abundances [95% CI (0.007, 0.19%)], similar to contamination ranges observed previously in mock communities (Lee et al., 2012; Esling et al., 2015). Two possible scenarios could result in the dependence of contaminant quantity and proportion on the number of cycles in the second step of PCR and the observation that the contaminants primarily map to amplicons that were sequenced in the same run. One possible explanation is that barcode-head primers used during the second step PCR reaction may be cross-contaminated, which may have resulted in the addition of the wrong barcode to a small proportion of amplicons in a given library. Low level cross-contamination of primers can occur during commercial oligonucleotide synthesis or handling of primers in the laboratory. A second explanation is that cross contamination of initial PCR products may have occurred in the laboratory between the first and second PCR reaction. Therefore, to minimize the impact of accidental contamination, we recommend that the number of cycles in step 2 should be kept to a minimum, i.e., five in this study. These results also reinforce the sentiment that healthy skepticism should be practiced when interpreting rare OTUs in a dataset (Reeder and Knight, 2009).

It is known that long sequences suffer from a bias in Illumina sequencing due to inefficient clustering on the Illumina flow cell (Bronner et al., 2009). We were particularly interested in exploring this bias across the entire dataset, because a broadly applicable multiplexing method should be robust to variation in gene target and amplicon length. Our 919 amplicon libraries of various phylogenetic and functional marker genes varied in length from approximately 290 to 720 bp (Figure 4, Supplemental Table 2.2). We observed large variations in the ratio of observed: expected library fraction [median = 0.79, 95% CI (0.103, 2.99)] that could be attributed to preparer (*p* < 2e-16), target gene (*p* < 2e-16) and target amplicon length (*p* = 1.4e-4). However, we did not detect a significant linear relationship between the target amplicon length and the ratio of observed: expected library fraction when gene target and preparer were included in the model (*p* = 0.14). Instead, there were singular instances in which the ratio of observed: expected library fraction was higher than others for gene target (*nxrB*, *p* = 0.032) and sample preparer (*p* ≤ 2e-16). We interpret this to indicate that stochastic inter-individual variability (i.e., experimental error) in library preparation played a larger role than amplicon length in determining the amount of data that could be assigned to each dataset. We infer that pooling amplicons in an equimolar manner is sufficient to return approximately equal read numbers for each target, unless empirical evidence exists that a specific gene target suffers significant and systematic bias.

In summary, we have developed a cost-saving approach based on two-step PCR barcoding that can be easily applied and adapted for any suitable target gene. Application of the universal library of barcode-head primers to a new gene or gene region of interest only requires the purchase of one pair of diagnostic primers with the 5′ head sequence instead of all combinations. Counting forward, reverse, and barcode-head primers, we used just over 350 relatively short primers to produce over 900 unique amplicon libraries. Producing these libraries using a standard approach would have required over 900 long fusion primer sets (i.e., more than 1800 primers in total). While we specifically tested this approach on the Illumina MiSeq platform and for microbial community analysis, this general barcoding approach can be easily modified for highly multiplexed amplicon sequencing on any sequencing platform and any gene or mutant gene library (van Opijnen and Camilli, 2013).

## Author contributions

AL and DB designed experiments with help of all other authors. CP, OK, BH, and RA performed experiments. CH, DB, CP, OK, BH, and RA analyzed data. CH, DB, and AL wrote the manuscript.

### Conflict of interest statement

The authors declare that the research was conducted in the absence of any commercial or financial relationships that could be construed as a potential conflict of interest.
