# Corrigendum: A flexible and economical barcoding approach for highly multiplexed amplicon sequencing of diverse target genes

**DOI:** 10.3389/fmicb.2016.00870

**Published:** 2016-06-06

**Authors:** Craig W. Herbold, Claus Pelikan, Orest Kuzyk, Bela Hausmann, Roey Angel, David Berry, Alexander Loy

**Affiliations:** Division of Microbial Ecology, Department of Microbiology and Ecosystem Science, Research Network Chemistry Meets Microbiology, University of ViennaVienna, Austria

**Keywords:** MiSeq, functional gene, 16S rRNA, amoA, nifH, dsrA, dsrB, nxrB

Reason for Corrigendum:

In the original article, we neglected to mention that Roey Angel, received funding from the Austrian Science Fund (FWF), grant number P25700-B20. The authors apologize for this oversight. This error does not change the scientific conclusions of the article in any way.

## Author contributions

All authors listed, have made substantial, direct and intellectual contribution to the work, and approved it for publication.

### Conflict of interest statement

The authors declare that the research was conducted in the absence of any commercial or financial relationships that could be construed as a potential conflict of interest.

