# Supplementary material for: A flexible and economical barcoding approach for highly multiplexed amplicon sequencing of diverse target genes
Source: Front Microbiol. 2015 Jul 16;6:731. doi: 10.3389/fmicb.2015.00731 (PMC4503924; doi:10.3389/fmicb.2015.00731)
Supplement: Supplementary file 1 [file Table1.DOCX]

**Table S1.1.** List of primers used and expected approximate target amplicon length. Primer binding sites (*E. coli* position) for 16S rRNA gene-targeted primers are indicated.

| Target gene | Target group | Forward primer sequence (5′-3′) | Reverse primer sequence (5′-3′) | Approximate amplicon length (bp),  including primer, head, and barcode | Annealing temperature (Cycle 1) | Reference |
| --- | --- | --- | --- | --- | --- | --- |
| *amoA* | Alpha subunit bacterial ammonium monooxygenase | GGGGHTTYTACTGGTGGT | CCCCTCKGSAAAGCCTTCTTC | 491 | 55 C | F- (Stephen et al., 1999)  R- (Rotthauwe et al., 1997) |
| *dsrA* | Alpha subunit bacterial-type reductive dissimilatory (bi)sulfite reductase | CACTGGAARCAYGGYG CACTGGAAGCACGGHG CATTGGAAGCATGGWG CATTGGAAACAYGGYG YACTGGAAGCATGGAG CATTGGAAGCAYGGCG CAGTGGGGATTCGGTG TATTGGAAGCACGGTG CATTGGAAACACGGCG TACTGGAAGCAYGGTG TACTGGAAACACGGCG | NNATRCARTGCATRCA NNAGACAGTRCATGCA NNAGGCAGYGCATGCA NNATRCAGTTCATGCA NNATGCAGTGKATGCA NNAGGCAATGCATGCA NNAGACATCTCATACA NNATRTAGTGCATGCA NNATGCAGTGSGTGCA NNAGACAATGCATGCA | 722 | 60 C-50 C touchdown | Pelikan unpublished |
| *dsrB* | Beta subunit bacterial-type reductive dissimilatory (bi)sulfite reductase | CAYACCCAGGGNTGG CAYACBCAAGGNTGG CATACDCAGGGHTGG CACACDCAGGGNTGG CACACDCAGGGNTGG CATACHCAGGGNTAY CAYACACAAGGATGG CACACCCAGGGWTTC CACACHCAGGGCTAT CACACBCAGGGMTAC CACACSCAGGGKTAY | CAGTTDCCRCARTACAT CAGTTACCRCAGAACAT CAGTTGSCGCAGAACAT CAGTTYCCGCAGAACAT CAGTTKCCACAGAACAT CAATTWCCACAGAACAT CAGTTGGCACAATACAT CAATTGCCGCAGAACAT CAATTTCCGCAGTACAT CAATTGGCACAGTACAT CAGTTAGCGCACATCAT CAGGCACCGCAGAACAT CAGTTGCCGCAAAACAT | 352 | 60 C-50 C touchdown | Modified from (Steger et al., 2011; Lever et al., 2013) |
| *nifH* | Nitrogenase subunit | GCIWTHTAYGGIAARGGIGGIATHGGIAA | ATIGCRAAICCICCRCAIACIACRTC | 394 | 52 C | (Ando et al., 2005) |
| *nifH* | Nitrogenase subunit | GCIWTYTAYGGIAARGGIGG | GCCATCATYTCICCIGA | 454 | 52 C | F - (Ueda et al., 1995)  R - (Marusina et al., 2001) |
| *nifH* | Nitrogenase subunit | TGYGAYCCIAAIGCIGA | GCCATCATYTCICCIGA | 358 | 52 C | (Marusina et al., 2001) |
| *nxrB* | Beta subunit nitrite oxidoreductase of *Nitrospira* and *Nitrospina* | TACATGTGGTGGAACA | CGGTTCTGGTCRATCA | 485 | 56 C | (Pester et al., 2014) |
| SSU rRNA | *Nitrospira* 16S rRNA gene region 534-1158 | GTGCCAGCMGCCGCGGTAA | CCCGTTMTCCTGGGCAGT | 661 | 56 C | F - (Walters et al., 2011)  R - (Maixner et al., 2006) |
| SSU rRNA | Bacterial 16S rRNA gene region 357-785 | CCTACGGGNGGCWGCAG | GACTACHVGGGTATCTAATCC | 464 | 55 C | (Herlemann et al., 2011) |
| SSU rRNA | Bacterial and archaeal 16S rRNA gene region 537-785 | CAGCMGCCGCGGTAATWC | TACNVGGGTATCTAATCC | 283 | 48 C | F- (Liu et al., 1997)  R - (Claesson et al., 2010) |
| SSU rRNA | Bacterial and archaeal 16S rRNA gene region 534-787 | GTGCCAGCMGCCGCGGTAA | GGACTACHVGGGTWTCTAAT | 292 | 52  56 | F - (Walters et al., 2011)  R - (Caporaso et al., 2011) |
| SSU rRNA | Eukaryotic 18S rRNA gene | GTGCCCTTCCGTCAATT | AGTTAAAAAGCTCGTAGTTG | 450-750 | 55 | (Lydolph et al., 2005) |

**Table S1.2.** Barcode sequences (5′-3′). 300 barcodes of a list of previously published 8-mer barcodes (Hamady et al., 2008) were used in this study.

| AACCAAGG | AACCGGTT | AACGATGG | AACGTAGG | AACGTTCG | AAGGAAGG | AAGGATCG | AAGGCGAT |
| --- | --- | --- | --- | --- | --- | --- | --- |
| AAGGTACG | ACACACTG | ACACCTCT | ACACGACT | ACAGAGTG | ACAGGAGT | ACAGTGAG | ACCATGGT |
| ACCTACCT | ACCTAGGT | ACGAACCT | ACGAAGGT | ACGACAAG | ACGACTTG | ACGTCATG | ACGTGAAG |
| ACGTGTTG | ACGTTGGT | ACTCACAG | ACTCCACT | ACTCTCTG | ACTCTGAG | ACTGACTG | ACTGAGAG |
| ACTGGACT | ACTGTCAG | ACTGTGTG | AGACACAG | AGACCTGT | AGACGTCT | AGACTCTG | AGACTGAG |
| AGAGACTG | AGGAGAAG | AGGATGGT | AGGTGATG | AGTCACTG | AGTCAGAG | AGTCCAGT | AGTCCTCT |
| AGTCGACT | AGTCTCAG | AGTGACAG | AGTGAGTG | AGTGCACT | AGTGGAGT | AGTGGTCT | AGTGTGAG |
| ATATGCCG | ATCCAACG | ATCCGGAT | ATCCTAGG | ATCCTTCG | ATCGATCG | ATCGTTGG | ATGGAACG |
| CAACCATG | CAACCTAG | CAACTCCT | CAACTGGT | CAAGACCT | CAAGAGGT | CACAACTG | CACACAGT |
| CACAGTGT | CACATCAG | CACATGTG | CACTACAG | CACTCTGT | CACTGAGT | CACTGTCT | CACTTCTG |
| CAGAAGTG | CAGACTGT | CAGAGTCT | CAGATCTG | CAGTACTG | CAGTAGAG | CAGTCAGT | CAGTCTCT |
| CAGTGACT | CAGTTCAG | CATCACCT | CATCCAAG | CATCCTTG | CATCGATG | CATCGTAG | CATGGTTG |
| CATGTGGT | CCAACCTT | CCAAGGAA | CCAATACG | CCATATGG | CCATCCAT | CCGGAATT | CCTAATGG |
| CCTACCAT | CCTACGAA | CCTACGTT | CCTATAGG | CCTATTCG | CCTTAAGG | CCTTCCAA | CCTTCCTT |
| CGAACGTT | CGAAGGAT | CGAATAGG | CGATCCAA | CGATCCTT | CGATGGAA | CGTACCTT | CGTACGAT |
| CGTAGGAA | CGTTCGTT | CGTTGCAA | CGTTGGAT | CTACACCT | CTACCAAG | CTACGTAG | CTAGTCCT |
| CTAGTGGT | CTCAACAG | CTCAAGTG | CTCACACT | CTCATCTG | CTCATGAG | CTCTACTG | CTCTCTCT |
| CTCTGACT | CTGAACTG | CTGACAGT | CTGACTCT | CTGAGACT | CTGAGTGT | CTGATGTG | CTGTCTGT |
| CTGTGAGT | CTGTTCTG | CTTCCATG | CTTCCTAG | CTTCGAAG | CTTCGTTG | CTTCTCCT | GAACCAAG |
| GAACCTTG | GAACGATG | GAAGGTTG | GAAGTCCT | GAAGTGGT | GACACACT | GACACTGT | GACAGTCT |
| GACATCTG | GACATGAG | GACTACTG | GACTAGAG | GACTGACT | GACTGTGT | GAGAACTG | GAGACAGT |
| GAGAGACT | GAGAGTGT | GAGTCACT | GAGTCTGT | GAGTGAGT | GAGTGTCT | GAGTTGAG | GATCCATG |
| GATCGAAG | GATCGTTG | GATCTCCT | GATCTGGT | GATGACCT | GATGAGGT | GATGGATG | GATGGTAG |
| GCAATAGG | GCATGGAA | GCATTACG | GCCGAATT | GCCGATAT | GCCGTAAT | GCCGTTAA | GCTAATCG |
| GCTACCTT | GCTACGAT | GCTATACG | GCTTCGTT | GGAACCAA | GGAAGGAA | GGAATACG | GGAATTGG |
| GGATATGG | GGATCCAT | GGATTAGG | GGTAATGG | GGTACCAT | GGTAGGAT | GGTATTCG | GGTTAAGG |
| GGTTCCTT | GGTTCGAT | GTACCTAG | GTACGAAG | GTACTCCT | GTAGACCT | GTCACTCT | GTCAGTGT |
| GTCATCAG | GTCTACAG | GTCTAGTG | GTCTCACT | GTCTCTGT | GTCTGTCT | GTCTTGAG | GTGACACT |
| GTGAGTCT | GTGATGAG | GTGTACTG | GTGTGTGT | GTGTTCAG | GTGTTGTG | GTTCACCT | GTTCCTTG |
| GTTCGATG | GTTGGTTG | GTTGTCCT | GTTGTGGT | TAATGCCG | TACCAACG | TACCGGAT | TACCTTCG |
| TACGATCG | TACGTACG | TAGGAACG | TAGGCCAT | TAGGCGTT | TAGGTAGG | TCACCACT | TCACGAGT |
| TCACTCTG | TCAGAGAG | TCAGGACT | TCCTTCCT | TCGATCCT | TCGTCAAG | TCGTCTTG | TCTCAGAG |
| TCTCCAGT | TCTCGACT | TCTCTCAG | TCTGACAG | TCTGAGTG | TCTGGAGT | TCTGGTCT | TGACGACT |
| TGACTCAG | TGAGACAG | TGAGTCTG | TGGAACCT | TGGACAAG | TGGAGTAG | TGGTCTAG | TGGTGAAG |
| TGGTTCCT | TGGTTGGT | TGTCAGTG | TGTCCACT | TGTCCTGT | TGTCGAGT | TGTCGTCT | TGTCTGAG |
| TGTGACTG | TGTGGTGT | TGTGTCAG | TGTGTGTG | TTCCGGAA | TTCCGGTT | TTCCTACG | TTCGAACG |
| TTCGATGG | TTCGTAGG | TTGGATCG | TTGGCCTT | TTGGCGAT | TTGGTTGG |  |  |

**Table S1.3.** Barcodes assigned to mock communities and cycle combination strategy. Primers targeted bacterial 16S rRNA gene region 357-785 from (Herlemann et al., 2011) (see Table S1.1)

| Mock community | Cycle variation | Barcode sequence 5′ to 3′ |
| --- | --- | --- |
| Uneven | 10:20 | CGAAGGAT |
| Even | 10:20 | CTCATGAG |
| Uneven | 15:15 | TTGGCGAT |
| Even | 15:15 | ACTCTGAG |
| Uneven | 20:10 | ACTCCACT |
| Even | 20:10 | ATCCTAGG |
| Uneven | 25:5 | GTTGTGGT |
| Even | 25:5 | GTCTAGTG |
| Uneven | 25:10 | CTTCGAAG |
| Even | 25:10 | CCTATTCG |
| Uneven | 30:5 | TGTCGAGT |
| Even | 30:5 | TCGTCAAG |
| Uneven | 30:10 | GAGAGACT |
| Even | 30:10 | GACTAGAG |
| Uneven | 35:5 | GTACCTAG |
| Even | 35:5 | CGTTCGTT |
| Uneven | 35:10 | CCTACCAT |
| Even | 35:10 | GTCTACAG |

**REFERENCES**

Ando, S., Goto, M., Meunchang, S., Thongra-ar, P., Fujiwara, T., Hayashi, H., and Yoneyama, T. (2005). Detection of nifHSequences in Sugarcane ( Saccharum officinarumL.) and Pineapple ( Ananas comosus[L.] Merr.). *Soil Science and Plant Nutrition* 51, 303–308. doi:10.1111/j.1747-0765.2005.tb00034.x.

Caporaso, J. G., Lauber, C. L., Walters, W. A., Berg-Lyons, D., Lozupone, C. A., Turnbaugh, P. J., Fierer, N., and Knight, R. (2011). Global patterns of 16S rRNA diversity at a depth of millions of sequences per sample. *Proceedings of the National Academy of Sciences* 108 Suppl 1, 4516–4522. doi:10.1073/pnas.1000080107.

Claesson, M. J., Wang, Q., O'Sullivan, O., Greene-Diniz, R., Cole, J. R., Ross, R. P., and O'Toole, P. W. (2010). Comparison of two next-generation sequencing technologies for resolving highly complex microbiota composition using tandem variable 16S rRNA gene regions. *Nucleic Acids Res* 38, e200. doi:10.1093/nar/gkq873.

Hamady, M., Walker, J. J., Harris, J. K., Gold, N. J., and Knight, R. (2008). Error-correcting barcoded primers for pyrosequencing hundreds of samples in multiplex. *Nat Meth* 5, 235–237. doi:10.1038/nmeth.1184.

Herlemann, D. P., Labrenz, M., Jürgens, K., Bertilsson, S., Waniek, J. J., and Andersson, A. F. (2011). Transitions in bacterial communities along the 2000 km salinity gradient of the Baltic Sea. *ISME J* 5, 1571–1579. doi:10.1038/ismej.2011.41.

Lever, M. A., Rouxel, O., Alt, J. C., Shimizu, N., Ono, S., Coggon, R. M., Shanks, W. C., Lapham, L., Elvert, M., Prieto-Mollar, X., et al. (2013). Evidence for microbial carbon and sulfur cycling in deeply buried ridge flank basalt. *Science* 339, 1305–1308. doi:10.1126/science.1229240.

Liu, W. T., Marsh, T. L., Cheng, H., and Forney, L. J. (1997). Characterization of microbial diversity by determining terminal restriction fragment length polymorphisms of genes encoding 16S rRNA. *Applied and Environmental Microbiology* 63, 4516–4522.

Lydolph, M. C., Jacobsen, J., Arctander, P., Gilbert, M. T. P., Gilichinsky, D. A., Hansen, A. J., Willerslev, E., and Lange, L. (2005). Beringian paleoecology inferred from permafrost-preserved fungal DNA. *Applied and Environmental Microbiology* 71, 1012–1017. doi:10.1128/AEM.71.2.1012-1017.2005.

Maixner, F., Noguera, D. R., Anneser, B., Stoecker, K., Wegl, G., Wagner, M., and Daims, H. (2006). Nitrite concentration influences the population structure of Nitrospira-like bacteria. *Environmental Microbiology* 8, 1487–1495. doi:10.1111/j.1462-2920.2006.01033.x.

Marusina, A. I., Boulygina, E. S., Kuznetsov, B. B., Tourova, T. P., Kravchenko, I. K., and Gal'chenko, V. F. (2001). A System of Oligonucleotide Primers for the Amplification of nifH Genes of Different Taxonomic Groups of Prokaryotes - Springer. *Microbiology* 70, 73–78. doi:10.1023/A:1004849022417.

Pester, M., Maixner, F., Berry, D., Rattei, T., Koch, H., Lücker, S., Nowka, B., Richter, A., Spieck, E., Lebedeva, E., et al. (2014). NxrB encoding the beta subunit of nitrite oxidoreductase as functional and phylogenetic marker for nitrite-oxidizing Nitrospira. *Environmental Microbiology* 16, 3055–3071. doi:10.1111/1462-2920.12300.

Rotthauwe, J. H., Witzel, K. P., and Liesack, W. (1997). The ammonia monooxygenase structural gene amoA as a functional marker: molecular fine-scale analysis of natural ammonia-oxidizing populations. *Applied and Environmental Microbiology* 63, 4704–4712.

Steger, D., Wentrup, C., Braunegger, C., Deevong, P., Hofer, M., Richter, A., Baranyi, C., Pester, M., Wagner, M., and Loy, A. (2011). Microorganisms with novel dissimilatory (bi)sulfite reductase genes are widespread and part of the core microbiota in low-sulfate peatlands. *Appl. Environ. Microbiol* 77, 1231–1242. doi:10.1128/AEM.01352-10.

Stephen, J. R., Chang, Y. J., Macnaughton, S. J., Kowalchuk, G. A., Leung, K. T., Flemming, C. A., and White, D. C. (1999). Effect of toxic metals on indigenous soil beta-subgroup proteobacterium ammonia oxidizer community structure and protection against toxicity by inoculated metal-resistant bacteria. *Applied and Environmental Microbiology* 65, 95–101.

Ueda, T., Suga, Y., Yahiro, N., and Matsuguchi, T. (1995). Remarkable N2-fixing bacterial diversity detected in rice roots by molecular evolutionary analysis of nifH gene sequences. *J. Bacteriol* 177, 1414–1417.

Walters, W. A., Caporaso, J. G., Lauber, C. L., Berg-Lyons, D., Fierer, N., and Knight, R. (2011). PrimerProspector: de novo design and taxonomic analysis of barcoded polymerase chain reaction primers. *Bioinformatics* 27, 1159–1161. doi:10.1093/bioinformatics/btr087.
