# Supplementary material for: A flexible and economical barcoding approach for highly multiplexed amplicon sequencing of diverse target genes
Source: Front Microbiol. 2015 Jul 16;6:731. doi: 10.3389/fmicb.2015.00731 (PMC4503924; doi:10.3389/fmicb.2015.00731)

Figure S1.1. Identification of contaminant OTUs in mock community. Each point represents an OTU that was observed in the mock community datasets. The radius of each point is proportional to the maximal relative abundance of the OTU observed across all mock datasets. The position on the x-axis shows the % identity of the OTU to its best hit among the sequences used in constructing the mock community. A) The position on the Y-axis shows the % identity to OTUs observed in other datasets that were sequenced in the same MiSeq run. B) The y-axis shows the maximal relative abundance of the shown OTU in non-mock datasets. (Data used in constructing this figure can be found in Supplementary Table S2.1.)

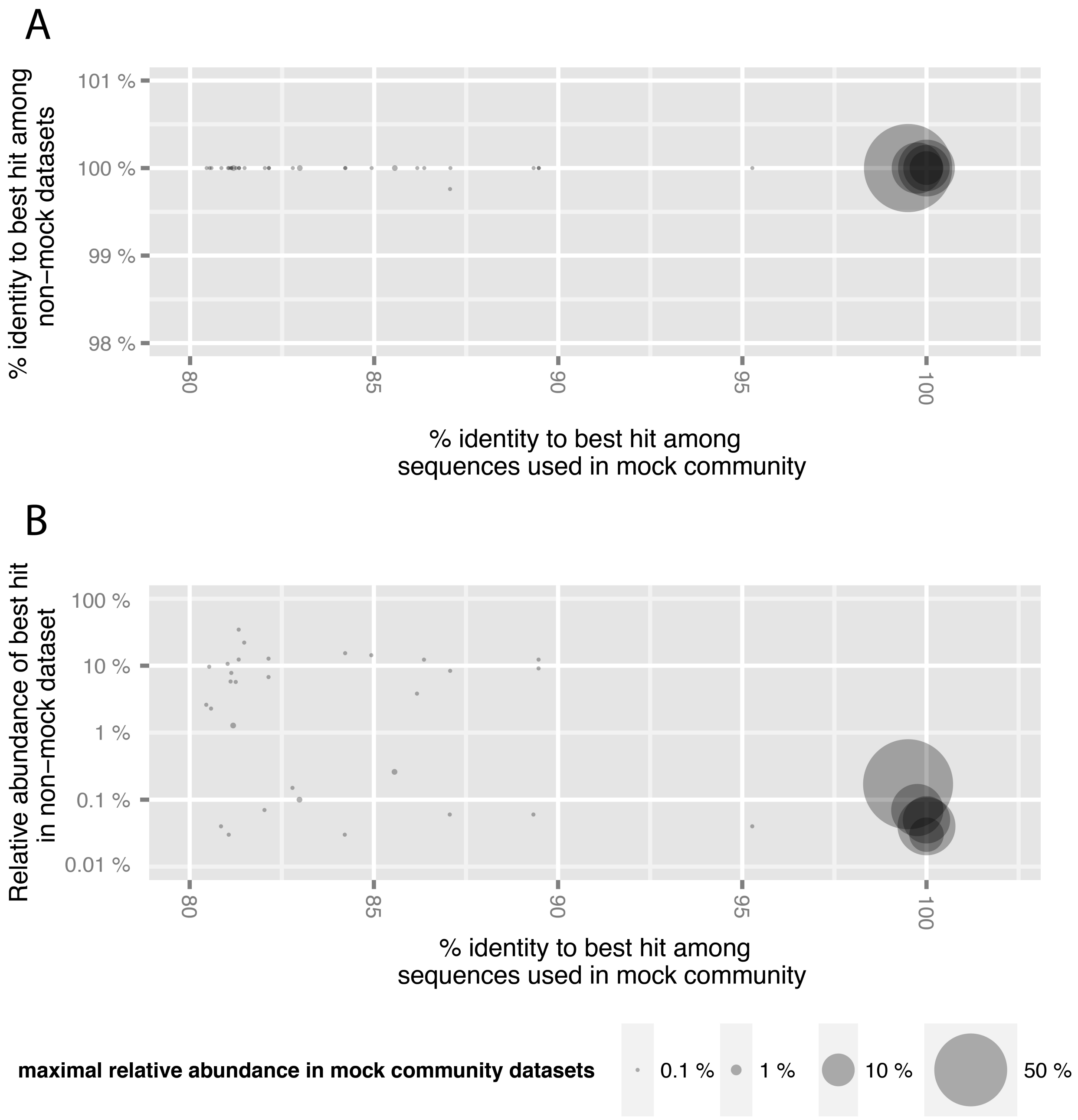

Supplement: Supplementary file 3 [file Image1.PDF]
